# Contribution to Reduce the Influence of the Free Sliding Edge on Compression-After-Impact Testing of Thin-Walled Undamaged Composites Plates

**DOI:** 10.3390/ma11091708

**Published:** 2018-09-13

**Authors:** Markus Linke, Juan Antonio García-Manrique

**Affiliations:** 1 Department of Automotive and Aeronautical Engineering, Hamburg University of Applied Sciences, 20099 Hamburg, Germany; 2 Department of Mechanical Engineering, Universitat Politécnica de Valencia, 46022 Valencia, Spain; jugarcia@mcm.upv.es

**Keywords:** Compression-After-Impact testing, Compression-After-Impact strength, carbon fiber reinforced plastics

## Abstract

Standard Compression-After-Impact test devices show a weakening effect on thin-walled specimens due to a free panel edge that is required for compression. As a result, thin-walled undamaged samples do not break in the free measuring area but near the free edge and along the supports. They also show a strength reduction due to the free edge which can become potentially relevant for very weakly damaged panels. In order to reduce the free edge influence on the measured strength, a modified Compression-After-Impact test device has been developed. In an experimental investigation with carbon fiber reinforced plastics, the modified device is compared with a standard device. It is shown that thin-walled undamaged specimens investigated with the modified device now mainly break within the free measuring area and no longer at the free edge and along the bearings as it is the case for standard test devices. The modified device does not cause a free edge weakening effect in comparison to standard devices. The modified device is therefore more suitable for determining the compression strengths of undamaged thin-walled composite plates.

## 1. Introduction

Thin-walled fiber-reinforced plastics, in particular, carbon fiber reinforced plastics (CFRP), are very susceptible to reductions in strength due to low velocity impact damages. In aeronautics, this strength loss has to be considered during construction by a sufficient damage tolerance leading to weight penalties. Therefore, it is important to understand the failure mechanisms of impact damaged composite structures in order to improve the damage tolerance of CFRP structures. 

The influence of impact damages on the static behavior is typically investigated by Compression-After-Impact (CAI) testing as the residual compressive strength is one major property that significantly decreases due to impact damages. It is a well standardized test (cp. AITM 1-0010 [1], ASTM D 7137 [2], BSS 7260 [3], EN 6038 [4], ISO 18352 [5], SACMA SRM 2R-94 [6]) where the residual strength of damaged composite plates is measured. The residual strength represents a structural strength value which results from the material properties of the plate, the structure of the CFRP, the induced damage mechanisms (such as interlaminar debonding, fiber breakage, matrix cracking, local-sublaminate buckling as well as their interactions) but also from the global geometrically non-linear behavior of the plate under pressure, in particular, as a result of geometric imperfections. Imperfections strongly influence the out-of-plane deformation during the whole compressive load application. Depending on the degree of out-of-plane deformation due to buckling, additional bending moments occur which lead to additional stress levels in the specimens. 

The severity of the damage is described by comparing the residual strength in CAI testing with the strength of undamaged specimens. The strength of undamaged specimens is determined either by the in-plane compression strength (e.g., according to ISO 14126 [7]) or by CAI testing (cp. [8,9]). 

If the in-plane compression strength of undamaged samples is compared to the residual CAI strength of damaged ones on the one hand, a structural strength value is compared with an in-plane material characteristic. The in-plane material characteristic ideally lacks completely an out-of-plane deformation since the free measuring length in the load direction is small with about 10 to 25 mm according to ISO 14126 [7]. However, the in-plane strength cannot be achieved in CAI testing as an out-of-plane deformation typically occurs during CAI testing resulting in an additional plate bending. The out-of-plane deformation is strongly influenced by geometric imperfections which can become relevant for thin-walled samples due to the free measuring size in the load direction of about 130 mm. As a result, the loss in strength is clearly overestimated. 

If the CAI strength is intended to be used for undamaged specimens to quantify the damage severity on the other hand, the measured CAI strength of the undamaged panel is usually reduced due to a free sliding edge which is required for compressing the panel. For a plate damaged with sufficient impact energy, this is generally unproblematic as the damage weakens the plate more than the free edge. The plate then breaks in the area of the damage. However, in the case of an undamaged or only very slightly damaged plate, the weakening of the plate due to the damage may be less than that caused by the free edge. For this reason, the fracture of such samples almost always occurs in the area of the free edge (cp. e.g., [10]). Consequently, the strength in CAI testing is generally underestimated for undamaged samples. This can be relevant also for very weakly damaged samples under specific circumstances. In these cases, standard CAI testing device leads to a systematic error.

Various modifications to CAI test devices are proposed in scientific literature. These differ primarily as a result of the different requirements that CAI testing equipment must meet.

Many articles deal intensively with the prevention of global buckling by the use of adequately modified CAI devices [10,11,12,13,14,15,16,17,18]. In principle, it is intended to first induce local effects such as local-sublaminate buckling due to delaminated layers in the damaged area. The local buckling results in further matrix cracks and interlaminar debonding finally leading to the collapse of the structure. Since the risk of global buckling is greatest in particular for thin-walled panels, the majority of these articles treats thin composite plates. 

Global buckling is basically shifted to higher buckling loads by two main measures. One reduces the free area in the CAI equipment. With the other, additional bearings are applied without changing sample geometry. Typically, the geometry still corresponds to the dimensions according to standardized CAI tests in this case.

The reduction of the free area is achieved either by applying double-sided stabilizing plates (cp. e.g., [10,13,14,15,17]) to the specimen called anti-buckling plates or by reducing the overall dimensions of CAI test samples through modifications of the equipment (cp. e.g., [11,12]). In both cases, the free measuring area is reduced which is accompanied by an increase in the global buckling load. Moreover, the influence of geometric imperfections is reduced so that out-of-plane deflections under the same load become smaller compared to the unstabilized plate. The stabilizing plates on the sample are either rectangular [10,13,14] or circular [17].

Furthermore, additional lateral supports are introduced on both sides of the samples in comparison to the standardized tests. The bearings are effective as edge knife simple supports. In [16], an additional narrow guide on both sides is applied between the simply supported lateral edges. It acts in the lateral direction over the entire measuring range. The continuous guide is positioned eccentrically so that the guide does not cover the damaged area. Four lateral guides are introduced according to [18] in such a manner that a gap to avoid inference with the damaged area is realized. Due to these guides, the global buckling load is increased. 

Research works also deal with the reduction of residual strength in CAI testing due to the potential misalignment with respect to the loaded edges [19]. CAI testing modifications are also reported in the case where the geometry of the specimens under consideration does not fit into the standardized ones (cp. [20] for tapered plates). 

Although it is reported in literature that failure in CAI testing occurs under certain conditions at the loaded supports [10], the influence of the weakening effect of the free specimen edge in CAI testing on the residual strength is hardly tackled. Only according to [10] a device is proposed which positions the weakening effect to the plate center. Therefore, the specimens no longer break at the supports but in the plate center. However, the strength reduction due to the free plate edge is not reduced as its length is not changed. 

This work deals with the free edge weakening effect of undamaged thin-walled plates in CAI testing. Usually the samples break in the area of the free edge at the bearing and not in the free measuring range. The resulting strength loss which is observed for undamaged samples can also become relevant for very weakly damaged panels as the weakening effect of the free edge can be greater than that of very low energy impact damages. As a result, the influence of strength reduction due to impacts is either not measurable for very weakly damaged panels or is subject to a high degree of uncertainty. In order to illustrate solutions to these difficulties, this work consequently aims at reducing the influence of the free edge in CAI testing on the measured strength of thin-walled samples. For this purpose, an existing CAI testing device is modified in such a way that undamaged samples no longer break in the area of the free edge along the bearings. The comparison with results obtained with a standard CAI device allows to estimate the weakening effect of the free edge. The work focuses on the problem of undamaged samples since the influence of the free edge can be determined more clearly with these than it is possible with damaged samples.

This article is subdivided into six sections. The introduction is followed by Section 2 where typical standards for CAI testing are described. Based on that, the argumentation for the selected modifications is illustrated leading to a modified CAI test device. In Section 3, the specimens and test campaign under investigation are described. The test results are illustrated in Section 4 and discussed in Section 5. Finally, the article concludes with Section 6.

## 2. Devices for Compression-After-Impact Testing and Test Device Modifications 

In aerospace engineering, two different types of fixtures for CAI testing are used. In principle, they differ by the supports at the loaded plate edges. Some CAI test devices exhibit a clamped support. And in contrast to this, other devices realize more or less simply supported edges at the loaded boundaries. In this article, CAI test devices of the former approach, i.e., with a realization of clamped supports at the loaded edges, are investigated. This is due to the fact that a clamped support is practically easier to be realized, in particular, if modifications to the CAI test device are intended to be made. 

**Figure 1:**
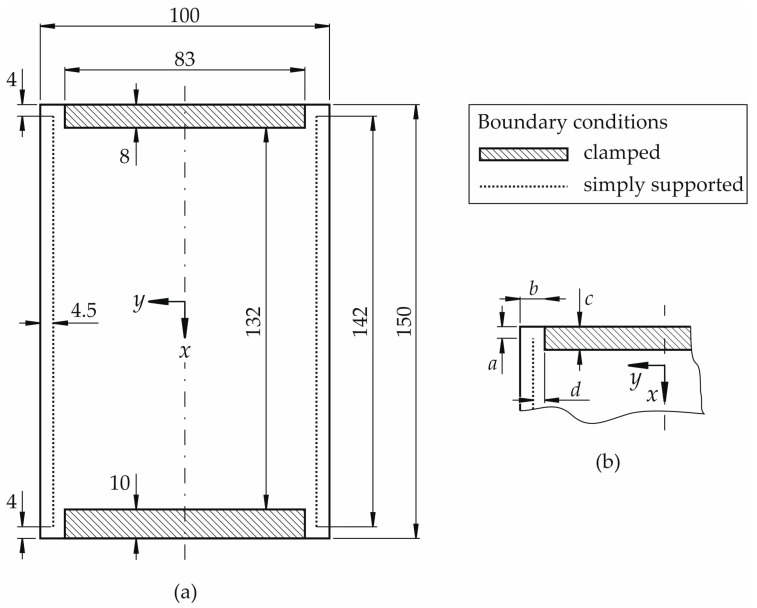
(**a**) Schematic fixture of composite plate (nominal dimension 100 mm × 150 mm) within typical CAI-standard test devices (cp. e.g., standards [1,5]) and (**b**) modified upper corner of CAI test device.

The standard test device under consideration for the investigation is the Instron “Airbus” Compression Impact Fixture (Instron GmbH, High Wycombe, UK) which agrees with the standards AITM 1-0010 [1] and ISO 18352 [5]. This device is called in the following CAI-standard device or briefly CAI-standard. The geometry and the plate fixture are schematically illustrated in Figure 1a. The nominal dimensions of the specimens to be tested amount to 100 by 150 mm^2^ indicated by the surrounding rectangle in Figure 1a. The shaded areas show the clamped part of the coupons. The clamping is achieved by putting specimens between to plane rigid walls of metal. The simply supported bearings are indicated by the dotted lines in the loading direction of the specimen which are parallel to the *x*-axis. They are applied in the same manner on both sides of the samples. The free measuring area is consequently about 91 by 132 mm^2^. The whole edge (100 mm) of the top part of the specimen is loaded whereas the bottom part is completely restrained and does not move. 

CAI testing using the CAI-standard for undamaged thin-walled composite specimens typically leads to failure at the top clamped boundary starting usually at the free edge of the specimens. Failure does not typically occur in the free range of the specimen like it is the case for damaged specimens. This is due to the fact that the weakening effect of the free edges is greater than the one of plate imperfections. E.g., geometric plate imperfections lead to a theoretical maximum plate bending in the middle of the free measuring area. 

Since the free edges at the two upper and two lower corners as well as their support along the width significantly reduce the failure load of undamaged specimens, these areas have been modified in a new CAI testing device which is called CAI-modified below. It is a CAI testing device where the boundaries at all four corners are changed in order to stiffen the free edge area. 

The free edges in the lower part of the device are not required for compression. Therefore, they are replaced by clamped supports. 

In the upper corners, the two free edges are stiffened by a change in the adjacent bearings. The stiffening effect is primarily caused by constraining the rotational degrees of freedom. This is due to the fact that plate rotations in this area induce additional bending stresses, thereby reducing the failure load. The main dimensions with a stiffening effect are exemplarily shown in Figure 1b. The shorter the free edge *a* is selected, the stiffer the plate becomes near the free edge. The edge with length *b* primarily acts as a simple support allowing a rotation around the global *y*-axis. Clamping consequently leads to the constraint of the rotational degree of freedom and thus to stiffening. The dimension *c* of the clamped support influences the rotational degrees of freedom around the *x*- and *y*-axis in the area where the lateral knife edge support acts. The further the clamping extends into the area between the lateral simple supports (indicated by dotted lines in the Figure 1a,b), the more the free edge area is stiffened. The latter effect increases with a decreasing distance *d*.

**Figure 2:**
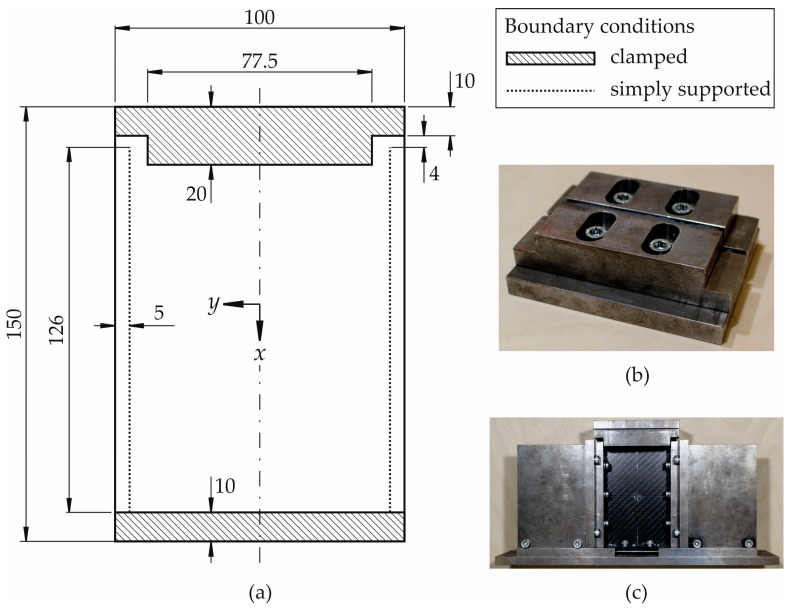
(**a**) Schematic fixture of composite plate (nominal dimension 100 mm × 150 mm) within modified CAI test device, (**b**) top part of modified CAI test device and (**c**) schematic fixture after modification with integrated sample.

Based on the described relations above between geometry respective constraints with free edge stiffening, the CAI-modified device is derived based on an already existing device manufactured in accordance with [1]. It is illustrated in a schematic sketch in Figure 2a. In the Figure 2b,c, photographs of the device are shown. As a certain length of the free edge is required for specimen compression in the *x*-direction, the dimension of the free edge has been maintained for about 4 mm (length *a* according to Figure 1b). But the specimen is now fully clamped along the whole loaded edge (length *b* according to Figure 1b). As the influence of the free plate edge can be reduced further by a clamped support that extends beyond the simply supported lines in the load direction (indicated by dotted lines in Figure 2a), the upper clamped support has been enlarged in the *x*-direction in the region between the simply supported lines compared to CAI-standard devices (length *c* according to Figure 1b). In order not to shorten too much the free measuring area, the support is enlarged by 2 mm in comparison to the standard geometry. The distance *d* according to Figure 1b is not reduced as the knife edge simple supports of the original device have been used.

In general, it has to be mentioned that this type of fixture is accompanied by a reduction of the free specimen area and therefore by an increase of the specimen stiffness which must be taken into account when comparing failure loads between the two different testing devices. 

## 3. Specimens and Test Campaign 

Specimens composed of CFRP are used. It is a unidirectional (UD) as well as a twill weave epoxy prepreg (SGL Technologies GmbH, Meitingen, Germany) which is processed by compression moulding through Clip Carbono (ClipCarbono.com, As Pontes, Spain) to plane plates with thickness of 2.2 mm. Both outer layers are composed of twill carbon weave (CW200-0/TW2/2 with 200 g/m^2^ according to [21]). Carbon UD-layers (CU150-0/SO with 150 g/m^2^ according to [21]) are positioned between them with 0° and 90° direction where the 0°-direction coincides with the *x*-axis according to the Figure 1a and Figure 2a. The layer set-up is symmetrical [twill weave, 0°, 90°, 0°, 90°, 0°, 90°]_s_. The matrix system is epoxy FT1021 according to [22]. The fiber volume content is estimated to 55% for twill weave layers and to 58% for UD layers. 

Specimens are investigated in an undamaged condition because it is intended to check whether the modifications of the CAI-modified device lead to failure within the free range of the specimens and not at the supports of the loaded edges where it is expected for the CAI-standard. Five specimens are tested each with the CAI-standard respective CAI-modified device. Sample geometry for CAI-standard testing agrees with the one according to [1], except for the width. In CAI testing with the CAI-modified device, the sample geometry is 98 mm × 150 mm and satisfied the tolerances specified by the AITM standard. The width is reduced by two millimeters in order to integrate the samples into the CAI-modified device without taking the risk of touching the lateral guides during testing as their distance amounts to 100 mm. Otherwise, an additional sample loading could result due to a prevented transversal displacement.

Measurements using the CAI-standard device are carried out with a universal material testing device (Shimadzu Autograph AG-X series with maximum 100 kN, Shimadzu Europe GmbH, Duisburg, Germany). The compression is displacement controlled with a rate of 0.5 mm/min. The displacement data is obtained from the optical strain system Shimadzu Digital Video Extensometer TRViewX (Shimadzu Europe GmbH, Duisburg, Germany). The data recording rate amounts to 1 Hz. The shortening of the samples is measured between two lines having a distance of 50 mm each to the plate center respective middle of the measuring range. 

The CAI-modified testing is performed with a servo-hydraulic material testing device (Schenck Hydropuls PSA, PZV 1865, with maximum 100 kN, Schenck AG, Darmstadt, Germany) equipped with Instron measuring instrumentation (Instron Deutschland GmbH Calibration Service, Darmstadt, Germany). The compression is load controlled with a rate of 0.25 kN/s leading to an averaged displacement rate of about 0.5 mm/min. Displacements are measured with the stereo pair camera digital imaging correlation (DIC) system ARAMIS (5 M sensor configuration, 2448 × 2050 pixel, Gom Gesellschaft für optische Messtechnik mbH, Braunschweig, Germany). Displacements are detected on the sample surface as far as both cameras have free view on the sample. The data recording rate is 1 Hz.

## 4. Test Results 

**Figure 3:**
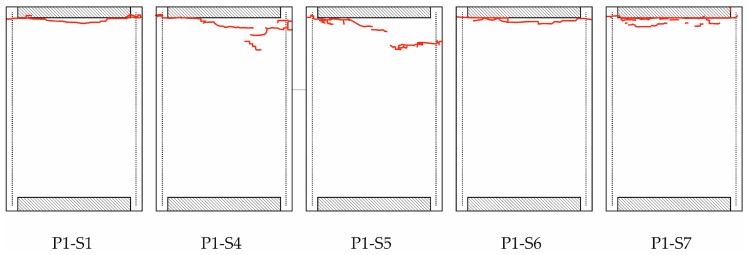
Observable fractures on the surface of the specimen front side tested with CAI-standard indicated by red lines (boundary conditions are described in Figure 1a) for the specimens P1-S1, P1-S4, P1-S5, P1-S6 and P1-S7.

**Figure A1:**
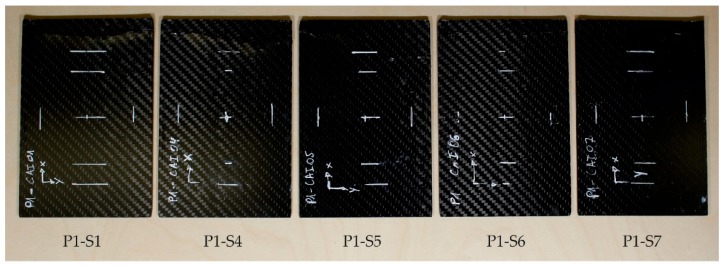
Front side of tested specimens P1-S1, P1-S4, P1-S5, P1-S6 and P1-S7 using CAI-standard device.

The fracture of the specimens tested with the CAI-standard device is illustrated in Figure 3. A photograph of the samples is shown in Appendix A, Figure A1. Concerning sample numbering, it has to be mentioned that the specimens are taken from a batch production of several panels. This batch production is the basis for several different investigations concerning CAI testing, among others, for impacted coupons, too. Therefore, the samples used here have a non-sequential numbering. This is also valid for the CAI-modified testing below. 

The red lines in Figure 3 indicate the fracture which can be observed on the surface of the specimens, i.e., on the side where the displacements are optically measured (called front side below). Although the red lines are not continuously connected from the left to the right side for the specimens P1-S4 and P1-S5, all specimens show fracture running through the whole specimen width. It is mainly a compression-shear fracture mode. Fracture occurs at the free edge of the CAI device and completely runs along the loaded edge at the clamped support for the specimens P1-S1, P1-S4, P1-S6 and P1-S7. Only specimen P1-S5 exhibits a fracture line which does not completely run along the support. It is a mixed fracture at the support and the free coupon area. The latter specimen also shows the highest failure stress for the CAI-standard testing (see Table 1). In order to cancel out slight deviations concerning the loaded specimen area, the failure stress σ_F_ is used as failure load. It is computed by the measured failure load divided by the specimen area. 

**Figure 4:**
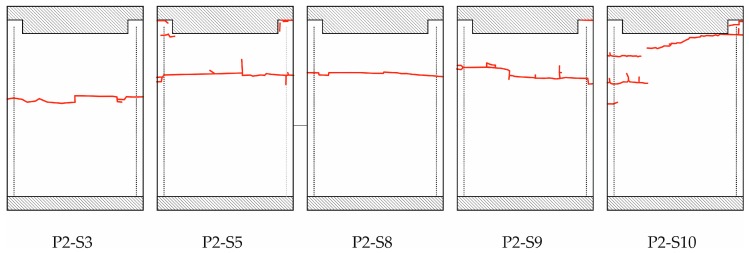
Observable failures on the surface of the specimen front side tested with CAI-modified (boundary conditions are described in Figure 2a) for the specimens P2-S3, P2-S5, P2-S8, P2-S9 and P2-S10.

**Figure 5:**
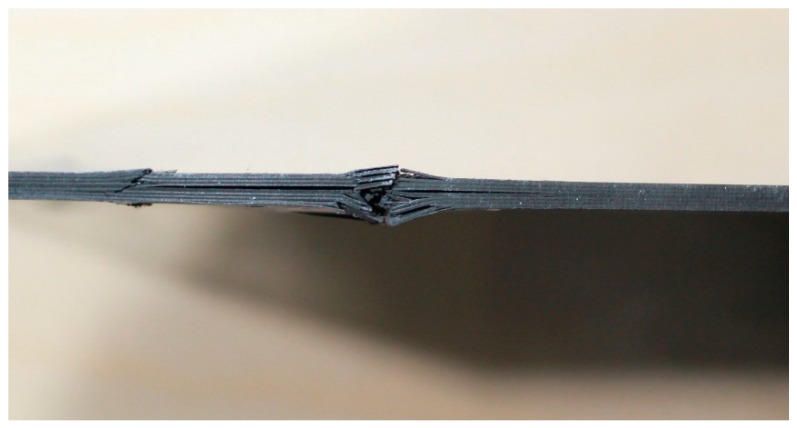
Illustrative kink zone and fracture, specimen P2-S10.

**Figure A2:**
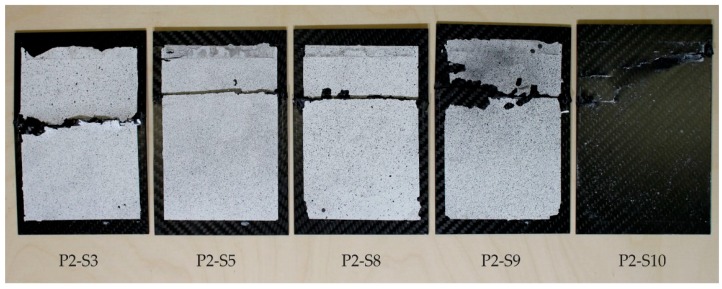
Front side of tested specimens P2-S3, P2-S5, P2-S8, P2-S9 and P2-S10 using CAI-modified device.

The fracture of the specimens tested with the CAI-modified device is illustrated in Figure 4. A photograph of the samples is shown in Appendix A, Figure A2. Again, the fracture on the surface of the front side is indicated by red lines. All specimens show fracture that runs along the whole width. The specimens typically exhibit compression-shear fracture modes that are combined with some delamination. The main failure modes are matrix cracks, delaminations and fiber breakage. A typical shear failure mode is observed, with interlaminar cracking in the middle part of the specimen. It can be noticed that some samples also exhibit a kink zone and fracture, as can be seen in Figure 5. The plastic deformation of the matrix followed by microbuckling evolves to kink zones, which provoke the fiber buckling, and the formation of two planes of fracture. 

This is also valid for specimen P2-S10 although the red lines are not completely connected on the surface. Fracture that is limited to the free range of the plate occurs for the specimens P2-S3, P2-S5, P2-S8 and P2-S9. Partially, fracture can be observed at the clamped support (for specimens P2-S5 and P2-S9) but these fractures are isolated and are not linked to the fracture line running through the free range of the plates. Contrary to that, specimen P2-S10 exhibits a mixed fracture, i.e., the fracture line runs at the clamped support into the free range of the plate. Its failure stress is lower than the ones for the specimens with fracture in the free range only (see Table 1). But the failure stress is in the range of specimen P1-S5 (CAI-standard testing) where the fracture line also runs from the support into the free range of the plate. 

**Figure 6:**
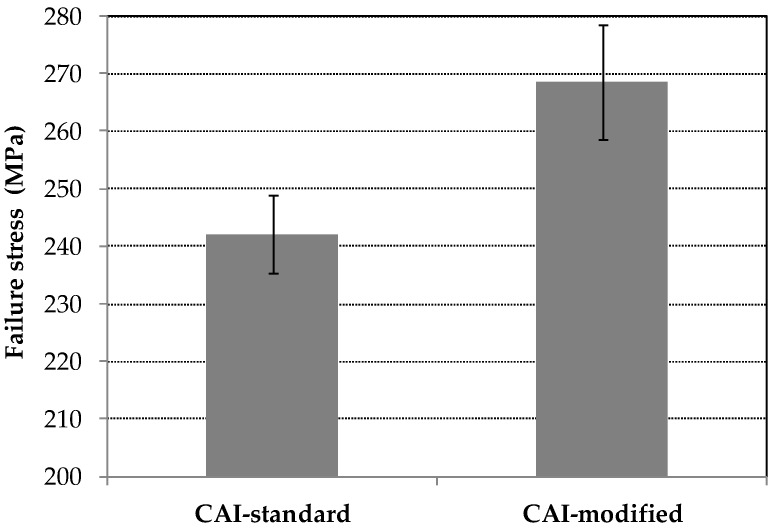
Failure stresses in CAI testing as well as the standard deviations for testing devices CAI-standard as well as CAI-modified.

The mean values of failure stress σ_F_ as well as the corresponding standard deviation s are computed based on the data according to Table 1. Results are illustrated in Figure 6. They amount for the CAI-standard testing device to

σ_F_ = 242.2 MPa, s = 6.9 MPa

and for the CAI-modified testing device to

σ_F_ = 268.6 MPa, s = 9.9 MPa.

**Figure 7:**
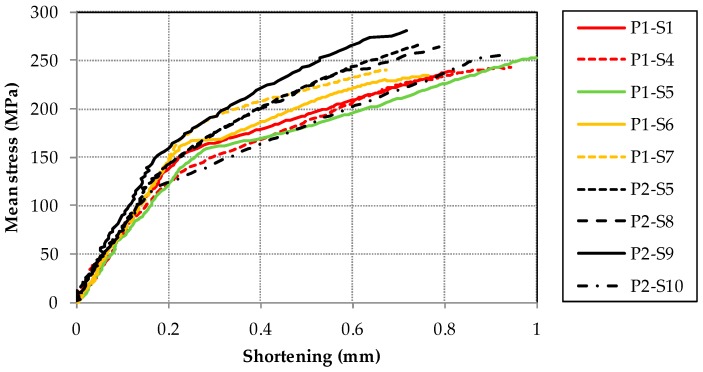
Mean stress versus shortening of specimens (for P2-S3 no digital imaging correlation data is available and the results for CAI-standard device are shown up to the maximum load).

The mean stress versus the shortening of the samples is shown in Figure 7. For the CAI-standard device, the shortening is directly measured according to Section 2 whereas the shortening concerning results of the CAI-modified device is computed based on DIC data related to the definition of the shortening of CAI-standard measurements. For the sake of simplicity, results of the CAI-modified respective CAI-standard device are illustrated with black respective colored lines. 

**Figure A3:**
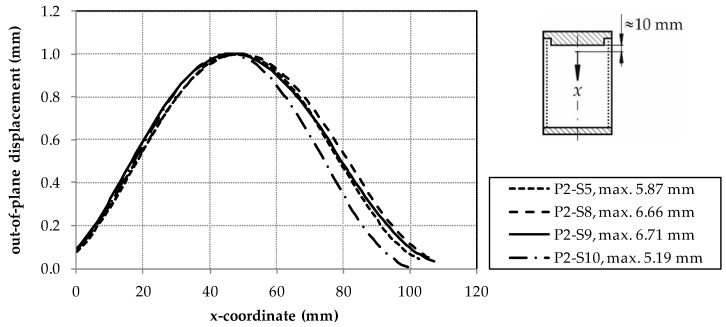
Normalized as well as maximum out-of-plane displacements of specimens investigated with CAI-modified device on the symmetrical line in global *x*-direction (cp. Figure 2a) starting near top bearing (for P2-S3 no digital imaging correlation data is available).

**Figure A4:**
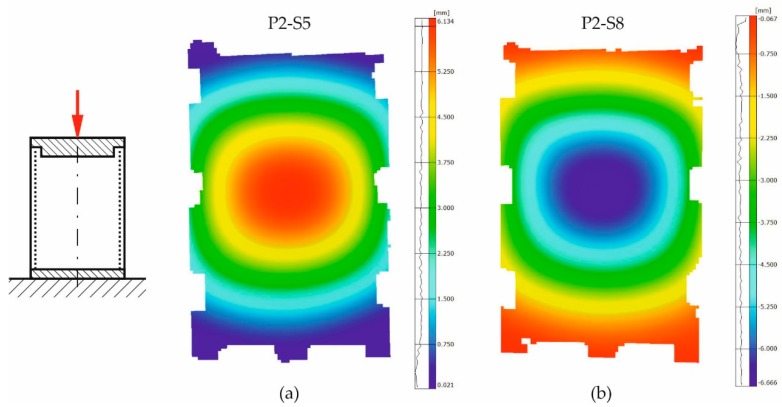
Qualitative illustration of out-of-plane displacement of specimen (**a**) P2-S5 and (**b**) P2-S8 measured with CAI-modified device.

**Figure A5:**
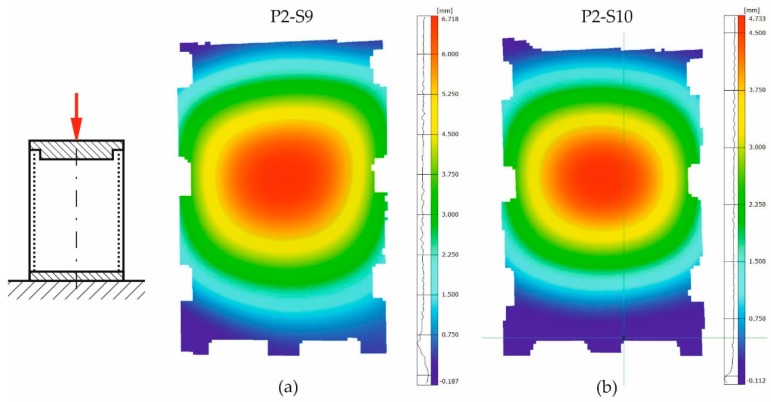
Qualitative illustration of out-of-plane displacement of specimen (**a**) P2-S9 and (**b**) P2-S10 measured with CAI-modified device.

In principle, all samples exhibit a linear trend between mean stress and shortening at the beginning of the measurement. According to DIC data of the CAI-modified testing, a global buckle starts to occur typically at 100 MPa which is then fully established of about 140 MPa in the plate center. From there on, the shape of the buckle remains constant. Only the absolute value of the out-of-plane displacement increases till failure (cp. out-of-plane displacements of measurements with the CAI-modified device in Appendix B in the Figure A3, Figure A4 and Figure A5). The failure of all samples occurs suddenly. 

## 5. Discussion of Results

The mean failure stress is significantly increased with the CAI-modified device compared to the standard one. The increase amounts to about 10% which is greater than the resulting standard deviations. The weakening effect of the free edge is consequently significant for CAI testing as the strength loss for low impact energies is in the order of magnitude of the increase. For quasi-isotropic thin-walled CFRP laminates with different layer set-ups, e.g., the decrease in CAI strength amounts to about 26 to 34% for impact energies of 0.5 J according to [23]. In [12] respective [14] a strength reduction of 5% (impact energy of 5.5 J) respective 7% (impact energy of 7.5 J) for quasi-isotropic thin-walled CFRP composites is reported. Due to that, the evaluation of the influence of damages due to very weak impact energy scenarios on the specimen strength should be estimated more precisely with the CAI-modified device. However, it has to be mentioned that the free measuring area between the supports is reduced, too, from 91 by 132 mm^2^ to 90 by 120 mm^2^. This comes along with a stiffening effect of the specimen. As a consequence, the bending loading of the plate is lowered compared to the CAI-standard test (if the same external load and equal geometric imperfections are assumed). Therefore, a thin-walled specimen investigated with the CAI-modified device will probably exhibit a higher failure load so that the increase of the mean failure stress is expected to be partly caused by the reduced free measuring area. However, this is probably of minor influence as similar quasi-isotropic CFRP laminates according to [12] exhibit a CAI strength difference of about 7% if the free measuring area respective lateral length is reduced to one half (compared to only 90% for the device discussed above). Moreover, results for similar fracture modes (according to P1-S5 and P2-S10) obtained from both devices exhibit failure stresses in the same stress range (cp. Figure 3 and Figure 4 as well as Table 1). Nevertheless, in order to facilitate a comparison where the influence of a reduced free measuring range is not existent, the measuring area should be increased to the size that agrees with typical standards. 

The standard deviation of the mean failure stress is increased in CAI-modified testing compared to CAI-standard from 2.8% to 3.7% of the mean value. However, this increase is mainly caused by an undesirable failure mode in CAI-modified testing where the rupture runs partially along the loaded upper support (cp. sample P2-S10 in Figure 4). If the sample P2-S10 is excluded, we obtain a standard deviation of 2.9% (for a mean failure stress of 271.8 MPa). This deviation is in the range of CAI-standard tests which should not be exceeded by a modified device. If CAI testing campaigns are carried out using the proposed CAI-modified device, the validation procedures of the selected samples should consequently conform to typical standards where unacceptable failure modes are discarded. For example, failure modes are typically not accepted if failure does not occur in the central free measuring area (between 20% to 80% scale of the free lateral measuring length).

The fractures lines of the specimens tested with the CAI-modified device are no longer positioned at the bearings but within the free measuring area also indicating that the weakening effect of the free edge no longer is relevant. But it has to be outlined that these lines are not positioned in or near the center of the free measuring area although their occurrence might be expected there due to the theoretical maximum bending loading. They occur in the upper part of the specimens. But no rupture is observed in the lower part. This phenomenon is unclear, in particular, as fracture modes of undamaged plates concerning CAI testing are not reported in open literature. However, there is a high probability that this is at least partly influenced by the device. The methodology followed is easily extensible to other combinations of fabrics or matrices, so its study is proposed for future work [24]. 

## 6. Conclusions

Due to the modification of the CAI test device at the loaded edge, the place of fracture of thin-walled CFRP samples no longer occurs at the clamped support only as it can be observed for standard CAI testing devices. The fracture lines usually run through the free range of the specimens. Furthermore, the failure stress is clearly increased indicating that the free edge of standard CAI devices weakens thin-walled samples in CAI testing. The intended aim of the research work is therefore achieved by the CAI-modified test device. The modified CAI testing device enables a more accurate measurement of CAI strengths of undamaged specimens. The modifications can be easily transformed to other CAI testing equipment as these usually work with similar loading edges. 

## Figures and Tables

**Table 1:** Failure loads and place of fracture (also indicated in Figure 3 and Figure 4) of different specimens for devices CAI-standard as well as CAI-modified with mean failure stress and standard deviation.

Denotation	CAI-Standard Failure Stress (Mpa)	Place of Fracture	Denotation	CAI-Modified Failure Stress (Mpa)	Place of Fracture
P1-S1	239.3	at support	P2-S3	276.9	free plate
P1-S4	242.7	at support	P2-S5	266.9	free plate
P1-S5	253.4	mixed	P2-S8	263.6	free plate
P1-S6	234.9	at support	P2-S9	279.8	free plate
P1-S7	240.4	at support	P2-S10	255.6	mixed
**Average:**	242.2		**Average:**	268.6	
**Deviation:**	6.9		**Deviation:**	9.9	

## Appendix A

The damaged front sides of the specimens tested with the CAI-standard respective CAI-modified device are shown in Figure A1 respective Figure A2.

## Appendix B

The normalized out-of-plane displacement on the symmetrical line according to Figure 2a is shown for specimens investigated with the CAI-modified device in Figure A3.

The out-of-plane displacement patterns of the specimens P2-S5, P2-S8, P2-S9 and P2-S10 shortly before sample failure occurs are illustrated in the Figure A4 and Figure A5. Only the surface area is shown which is in view of both cameras of the DIC measuring device. The gaps are caused by screws and shadows of the surrounding.
